# Supplementary material for: Enclosure in Combination with Mowing Simultaneously Promoted Grassland Biodiversity and Biomass Productivity
Source: Plants (Basel). 2022 Aug 4;11(15):2037. doi: 10.3390/plants11152037 (PMC9370151; doi:10.3390/plants11152037)
Supplement: Supplementary file 1 [file plants-11-02037-s001.zip › plants-1833252-supplementary.pdf]

# *Supplementary Material*

## Supplementary Tables

**Table S1**. The importance values of plant species surveyed in experimental plots T1 through T5.

| Functional group | Species | T1 | T2 | T3 | T4 | T5 |
|---|---|---|---|---|---|---|
| Poaceae | *Leymus chinensis* | 0.681 | 0.3 | 0.6 | 0.7 | 0.04 |
| Poaceae | *Poaceae hierochloeglabra* | 0.004 | 0.02 | | | 0.1 |
| Poaceae | *Stipa Baicalensis* | 0.005 | 0.02 | 0.01 | 0.02 | 0.01 |
| Poaceae | *Poa sphondylodes* | 0.01 | 0.01 | 0.01 | | 0.01 |
| Poaceae | *Achnatherum sibiricum* | | 0.02 | | | 0.06 |
| Poaceae | *Achnatherum sibiricum* | | 0.01 | 0.01 | | 0.05 |
| Poaceae | *Koeleria cristata* | | 0.02 | 0.02 | | 0.05 |
| Poaceae | *Helictotrichon schellianum* | | | | | 0.01 |
| Poaceae | *Festuca ovina* | | 0.01 | 0.03 | | 0.09 |
| Sedge | *Carex spp* | 0.33 | 0.13 | 0.09 | 0.06 | 0.16 |
| Sedge | *Carex pediformis* | | 0.17 | | | 0.06 |
| Legume | *Vicia amoena* | 0.01 | | | | |
| Legume | *Astragalus melilotoides* | 0.17 | 0.03 | | | |
| Legume | *Gueldenstaedtia verna* | | 0.03 | | | 0.01 |
| Legume | *Oxytropis myriophylla* | | | | | 0.01 |
| Legume | *Astragalus adsurgens* | | | | | 0.02 |
| Legume | *Thermopsis lanceolata* | 0.02 | 0.01 | 0.02 | | |
| Forb | *Artemisia commutata* | 0.02 | | | | 0.01 |
| Forb | *Potentilla bifurca* | 0.04 | 0.02 | 0.01 | | 0.01 |
| Forb | *Galium verum* | | 0.04 | | | 0.03 |
| Forb | *Potentilla tanacetifolia* | 0.02 | 0.02 | 0.01 | | 0.03 |
| Forb | *Fallopia convolvulus* | 0.04 | | | | |
| Forb | *Taraxacum mongolicum* | 0.01 | | 0.01 | | 0.04 |
| Forb | *Axyris amaranthoides* | 0.02 | | | | |
| Forb | *Heteropappus altaicus* | | | | 0.07 | |
| Forb | *Scorzonera divaricata* | 0.01 | | | | 0.01 |
| Forb | *Tephroseris kirilowii* | | 0.01 | | | |
| Forb | *Artemisia frigida* | | 0.01 | 0.01 | 0.02 | 0.02 |
| Forb | *Artemisia laciniata* | | 0.07 | 0.09 | 0.06 | 0.17 |
| Forb | *Serratula komarovii* | | 0.03 | | | 0.02 |
| Forb | *Iris ventricosa* | | 0.03 | | | 0.02 |
| Forb | *Adenophora stricta* | | 0.03 | | | 0.01 |
| Forb | *Allium bidentatum* | | 0.04 | 0.06 | 0.05 | 0.06 |
| Forb | *Pulsatilla turczaninovii* | | 0.09 | 0.01 | | 0.07 |
| Forb | *Allium tenuissimum* | | 0.02 | 0.02 | 0.04 | 0.02 |

| | | | | | |
|---|---|---|---|---|---|
| Forb | *Artemisia dracunculus* | 0.04 | | | |
| Forb | *Thalictrum squarrosum* | 0.09 | | | 0.03 |
| Forb | *Cymbaria daurica* | | 0.02 | | |
| Forb | *Lepidium apetalum* | | 0.04 | 0.02 | 0.01 |
| Forb | *Sibbaldia adpressa* | | 0.01 | | |
| Forb | *Chenopodium acuminatum* | | 0.01 | | 0.03 |
| Forb | *Dianthus chinensis* | | 0.01 | | |
| Forb | *Orostachys fimbriatus* | 0.02 | 0.04 | 0.05 | 0.01 |
| Forb | *Lychnis sibirica* | | 0.02 | | |
| Forb | *Dontostemon micranthus* | | 0.01 | | 0.02 |
| Forb | *Allium ramosum* | | 0.03 | 0.03 | 0.02 |
| Forb | *Convolvulus ammannii* | | 0.03 | 0.04 | |
| Forb | *Thalictrum petaloideum* | | | | 0.03 |
| Forb | *Bupleurum scorzonerifolium* | | | | 0.01 |
| Forb | *Iris tigridia* | | | | 0.02 |
| Forb | *Carpesium abrotanoides* | | | | 0.01 |
| Forb | *Ixeris polycephala* | | | | 0.01 |
| Forb | *Potentilla verticillaris* | | | | 0.01 |
| Forb | *Filifolium sibiricum* | | | | 0.01 |
| Forb | *Potentilla acaulis* | | | | 0.07 |

**Table S2**. Single linear regression models fitted to plant aboveground biomass in response to plant diversity index under field treatments. NS means not significant.

| Treatment | Index | Intercept | Slope | $R^2$ | Significance |
|---|---|---|---|---|---|
| T1 | Alatalo | 316.63 | -126.28 | 0.11 | NS |
| T1 | Margalef | 284.69 | -36.08 | 0.05 | NS |
| T1 | Patrick | 284.35 | -4.6 | 0.03 | NS |
| T1 | Pielou | 306.56 | -156.82 | 0.26 | NS |
| T1 | Shannon | 292.36 | -61.8 | 0.17 | NS |
| T1 | Simpson | 275.24 | -52.93 | 0.07 | NS |
| T2 | Alatalo | 487.19 | -384.53 | 0.21 | NS |
| T2 | Margalef | 384.85 | -49.6 | 0.21 | NS |
| T2 | Patrick | 447.98 | -11.88 | 0.34 | NS |
| T2 | Pielou | 542.82 | -407.04 | 0.7 | ** |
| T2 | Shannon | 472.59 | -109.47 | 0.62 | ** |
| T2 | Simpson | 546.99 | -386.05 | 0.64 | ** |
| T3 | Alatalo | -24.26 | 368.34 | 0.37 | NS |
| T3 | Margalef | 126.29 | 20.44 | 0.14 | NS |
| T3 | Patrick | 120.14 | 3.47 | 0.16 | NS |
| T3 | Pielou | 55.99 | 176.99 | 0.17 | NS |
| T3 | Shannon | 90.69 | 49.49 | 0.23 | NS |
| T3 | Simpson | 66.87 | 156.44 | 0.31 | NS |
| T4 | Alatalo | 225.97 | -94.63 | 0.06 | NS |
| T4 | Margalef | 240.91 | -57.27 | 0.39 | NS |
| T4 | Patrick | 251.89 | -9.25 | 0.35 | NS |
| T4 | Pielou | 225.34 | -84.71 | 0.26 | NS |
| T4 | Shannon | 225.25 | -41.26 | 0.36 | NS |
| T4 | Simpson | 221.46 | -86.25 | 0.31 | NS |
| T5 | Alatalo | 101.2 | -24.29 | 0.02 | NS |
| T5 | Margalef | 56.74 | 7.48 | 0.04 | NS |
| T5 | Patrick | 11.35 | 3.51 | 0.12 | NS |
| T5 | Pielou | 122.64 | -45.64 | 0.03 | NS |
| T5 | Shannon | 91.06 | -2.54 | 0 | NS |
| T5 | Simpson | 101.24 | -18.97 | 0 | NS |
